# The Relation between the Physician and Pharmaceutist

**Published:** 1872-09

**Authors:** Th. Schumann

**Affiliations:** Pharmaceutist, Atlanta, Georgia


### THE RELATION BETWEEN THE PHYSICIAN AND PHARMACEUTIST.

(*REPLY TO Z. T. DANIEL, M.D., EUFAULA, ALABAMA.*)

BY TH. SCHUMANN, PHARMACEUTIST, ATLANTA, GEORGIA.

Dr. Daniel publishes in the August number of the ATLANTA MEDICAL AND SURGICAL JOURNAL a reply to a little paper contributed to the May number of the JOURNAL, upon this subject, to which I wish to make the following answer.

If I understand Dr. Daniel's reply aright, it culminates in these points:

1. As long as you have anything to do with patent medicines, I will have nothing to do with you. Begone from me!

2. If education in the direction suggested is to improve the state of affairs, Th. Schumann, and others who may be of the same opinion with him, must attend to it; the medical profession is not interested in that business.

It would be a great mistake to believe that only the medical profession is disgusted with the patent medicine business. It is a matter of course that physicians should be opposed to it, as it injures their interests; but many others, and a great many pharmaceutists, are also disgusted with it. I never dreamed of using anything of that kind for myself or family, and thousands, with me, would not do it; but others do use them, and call for them at the drug-store. And what shall the druggist do, if he has a family to support? In Rome, he has to do as the Romans do; and under existing circumstances, he can not afford to abolish this branch of the trade entirely, as long as such a measure would not be generally adopted. If, however, Dr. Daniel has never put his foot into a drug-store where this peculiar trade has not been pursued, it only shows that there are drug-stores which Dr. Daniel has not seen. There are certainly not many drug-stores in the United States without a full assortment of patent medicines; but still there are some in the larger cities—about half a dozen or a dozen in New-York—where no patent medicine is kept. Sure enough, only German pharmaceutists could venture to do business in that manner, and I dare say they are diminishing as fast as the German population of these cities becomes Americanized.

In England, we find exactly the same usages and abuses. In France, the authorities hold a certain control over these nostrums. The Académie de Médecine examines the articles, and gives certificates of approval; and therefore the French patent medicines can be prescribed, and are prescribed by physicians, and bear mostly a very different appearance from the English, and especially the American, patent medicines. The French patent medicines mostly propose to give a certain recognized remedy in a more convenient or palatable form: for instance, balsam copaiba and similar remedies in capsules; or protoxide of iron in pills protected against the influence of oxygen by a certain varnish; charcoal in a more active and palatable form, etc.

In Germany, the sale of any patent medicine is prohibited; in fact, there are hardly any in existence—perhaps a dozen in all, without anything like a general sale. None are kept in drug-stores; only some general and fancy stores keep them as a kind of contraband. Pharmaceutists do not sell anything but medicines and chemicals belonging to the pharmacopœia, either for ordinary use by the public or for prescriptions, and a few cosmetics of their own manufacture—very little of the manufacture of the regular perfumers. These are the facts in regard to the pharmaceutists in Germany. They are just as bitterly opposed to the sale of nostrums and arcanums as physicians themselves, and as any sensible man should be.

The above shows, at least, that all druggists are not in favor of the patent medicine trade; and I would add, by my own experience, that many American pharmaceutists would with pleasure do away with all the patent medicines in their stores, and willingly confine themselves to the legitimate prescription and general drug business, but can not do it as long as others continue that trade. Those who would do so are certainly the better educated in pharmaceutical science; while men who never devoted themselves to study, and who only pursue the *drug trade* like any other *trade*, can not be expected to do so.

I can, therefore, only repeat what I said in the May number—educate the pharmaceutical profession, and protect them against impostors by law. If only educated pharmaceutists could keep drug-stores, or serve as drug clerks—if the trade in medicines was the exclusive privilege of the educated pharmaceutists—matters would stand differently: better for the pharmaceutist, better for the physician, and better for the public at large.

But, then, there is an intermediate link between the prescription and the patent medicine, which smells very strongly of the latter. I mean the uncounted number of elixirs, etc., with which the drug market is flooded. Does not the physician compromise himself when he prescribes Mr. A.'s "Elixir of Calisaya," or Messrs. B., C. & Co.'s "Elixir of Bromide of Potassium"? There is a difference between these preparations and "Ayers Sarsaparilla," or "Osgood's Cholagogue," but very little indeed.

Dr. Daniel seems to think regulation by law an absurd idea. Let us see, however, how things stand in other countries.

In England, as above mentioned, the same usages and abuses exist. The chemist keeps in his shop drugs, chemicals, perfumery, cutlery, crockery, toys, and everything he can sell. The grocer keeps epsom salts, laudanum, paregoric, sugar of lead, sulphate of zinc, etc.; but he does not know anything about the properties of his goods, nor does he care to know. In Germany and France, only educated pharmaceutists can sell drugs and medicines, or put up prescriptions. A pharmaceutist who passed the last examination, and complied with the necessary requirements, stands at the head of every pharmacy, whether he is the proprietor or not; and should the proprietor himself not be the examined pharmaceutist of the establishment, the supervisor (that is, the pharmaceutist) has the decision of all questions not of a purely financial character, and, of course, all the responsibility. Assistant pharmaceutists also have to pass an examination before they can leave apprenticeship and put up prescriptions; and young men are not even admitted to apprenticeship without having shown the necessary degree of education. Should any pharmacy not have an examined pharmaceutist among its employés, it would be closed by the authorities until such an examined manager or supervisor could be provided for.

Now, would it be so very impossible to institute a similar law in this country? I can not believe it. In fact, similar regulations have been enacted in Maryland, Pennsylvania, Rhode-Island, and perhaps in other States, and lately, after hard and prolonged struggles, in New-York. I am confident the day is not very far off when most of the States of the Union will have similar laws. The American Pharmaceutical Association works faithfully in that direction, and a great many well-thinking pharmaceutists, physicians, and laymen also. Of course, it is far easier to say, "Physicians have no desire to associate with you until you lend a helping hand to cut down this national monster," or, "It becomes Mr. Schumann and his colaborers to educate these unfortunate ones," than to join those who work for the advancement of civilization in this direction; but one man can do but very little, while many associated, or a class or classes of citizens, could do a great deal. Look at those States above mentioned, where pharmacy laws exist. Should Georgia or Alabama not be able to do the same? or is such a law against our ideas of liberty? Is liberty in Georgia or Alabama so little under the control of law as to admit of no hope in this direction?

I would most respectfully ask Dr. Daniel to join me, and, instead of ridiculing my efforts, weak as they may be, become a colaborer in the cause of promoting sound ideas on this subject. If all sensible men, especially all physicians, would be on the side of the pharmaceutist, *as they should be*, the war would already be half done; and I take the liberty to expect Dr. Daniel, in future, on my side of the question.

